# End Users' Perspectives on the Quality and Design of mHealth Technologies During the COVID-19 Pandemic in the Philippines: Qualitative Study

**DOI:** 10.2196/41838

**Published:** 2023-04-20

**Authors:** Aldren Gonzales, Razel Custodio, Marie Carmela Lapitan, Mary Ann Ladia

**Affiliations:** 1 Medical Informatics Unit College of Medicine University of the Philippines Manila Manila Philippines; 2 National Telehealth Center National Institutes of Health University of the Philippines Manila Manila Philippines; 3 Institute of Clinical Epidemiology National Institutes of Health University of the Philippines Manila Manila Philippines

**Keywords:** mHealth, COVID-19, pandemic, digital health, mobile health, end-user engagement, focus group, technology implementation, Philippines, technology use, privacy, user engagement

## Abstract

**Background:**

The COVID-19 pandemic has expanded the use of mobile health (mHealth) technologies in contact tracing, communicating COVID-19–related information, and monitoring the health conditions of the general population in the Philippines. However, the limited end-user engagement in the features and feedback along the development cycle of mHealth technologies results in risks in adoption. The World Health Organization (WHO) recommends user-centric design and development of mHealth technologies to ensure responsiveness to the needs of the end users.

**Objective:**

The goal of the study is to understand, using end users’ perspectives, the design and quality of mHealth technology implementations in the Philippines during the COVID-19 pandemic, with a focus on the areas identified by stakeholders: (1) utility, (2) technology readiness level, (3) design, (4) information, (5) usability, (6) features, and (7) security and privacy.

**Methods:**

Using a descriptive qualitative design, we conducted 5 interviews and 3 focus group discussions (FGDs) with a total of 16 participants (6, 37.5%, males and 10, 62.5%, females). Questions were based on the Mobile App Rating Scale (MARS). Using the cyclical coding approach, transcripts were analyzed with NVivo 12. Themes were identified.

**Results:**

The qualitative analysis identified 18 themes that were organized under the 7 focus areas: (1) utility: use of mHealth technologies and motivations in using mHealth; (2) technology readiness: mobile technology literacy and user segmentation; (3) design: user interface design, language and content accessibility, and technology design; (4) information: accuracy of information and use of information; (5) usability: design factors, dependency on human processes, and technical issues; (6) features: interoperability and data integration, other feature and design recommendations, and technology features and upgrades; and (7) privacy and security: trust that mHealth can secure data, lack of information, and policies. To highlight, accessibility, privacy and security, a simple interface, and integration are some of the design and quality areas that end users find important and consider in using mHealth tools.

**Conclusions:**

Engaging end users in the development and design of mHealth technologies ensures adoption and accessibility, making it a valuable tool in curbing the pandemic. The 6 principles for developers, researchers, and implementers to consider when scaling up or developing a new mHealth solution in a low-resource setting are that it should (1) be driven by value in its implementation, (2) be inclusive, (3) address users’ physical and cognitive restrictions, (4) ensure privacy and security, (5) be designed in accordance with digital health systems’ standards, and (6) be trusted by end users.

## Introduction

The COVID-19 pandemic has spurred innovative solutions from all sectors to respond to the public health crisis. This includes the application of mobile health, or mHealth, which refers to the use of mobile wireless technologies in health for public health measures and surveillance that have attracted more attention from the public [1-3]. More specifically, mHealth technologies have been used for early detection, faster screening, patient monitoring, information sharing, education, and treatment management in response to the COVID-19 outbreak [4].

In the Philippines, the rapid expansion of digital technology was evident during the first 2 quarters of 2021 when the government rolled out a number of mHealth apps to aid in contact tracing, communicate COVID-19–related information, and monitor the health conditions of the general population [5]. In addition to contact training tools, various local government units (LGUs) and private organizations also implemented mHealth technologies to facilitate virtual health consultation (ie, telehealth) [6], local and international travel [7], monitoring of symptoms [8], setting of vaccine appointments [9], and health and services information provision (eg, helpline, chatbot) [10], among others. Although there are no data available to measure adoption, the policies and guidelines set by the government [11,12] and the recognition of mHealth’s potentials promoted a digital approach to public health measures.

The rapid expansion of mHealth also made clear some gaps in its development and implementation. As we grappled with the pandemic, computer programmers prioritized releases to quickly deploy digital platforms. Consequently, despite the growing demand for mHealth in the Philippines, the absence of comprehensive frameworks and development guidelines leads to digital health tool administration silos and uncertainties.

Although deemed important, anecdotes from Filipino developers consulted noted that the rapid development resulted in limited end-user engagement to define requirements and collect feedback along the development cycle. The recognized lack of user engagement poses risks in adoption, because many apps fail to meet the requirements of their target users [13,14]. Ignoring user engagement can also result in overengineering solutions before having a good understanding of user needs [15] and eventually cause delays by needing to redesign features at the later stage of development or during roll-out [16].

User-centric development is considered important toward meaningful use and successful implementation [17]. Recognizing the importance of this approach, the World Health Organization (WHO) recommends integrating it into the life cycle of mHealth products to ensure effective results [18]. Various studies have likewise highlighted the importance of the user-centric design and development of mHealth technologies. Its application has shown positive results in (1) eliciting and integrating requirements critical to end users, (2) recognizing potential challenges, (3) verifying the workflow, and (4) designing information architecture; all these can contribute to improving adoption and resulting in improvements in the intended outcomes [14,19-22]. The user engagement approach has also resulted in the identification of user preferences in the privacy and security features of COVID-19 mHealth apps [23].

The gaps in development as well as in continued development and use of mHealth in the Philippines highlight the necessity to ensure that these tools are responsive to the needs of the end users. Recognizing the value of user engagement, the goal of this study is to understand the design and quality of mHealth technology implementations in the Philippines during the COVID-19 pandemic from the end users’ perspectives. Based on the results, we present user-centric design and implementation considerations for innovators, developers, and researchers in creating or scaling up mHealth, with particular attention paid to resource-limited settings.

## Methods

### Context

This study forms part of the rapid assessment of mHealth technologies in the Philippines project commissioned by the Philippines Department of Health – Health Technology Assessment Council (DOH-HTAC) to gain more understanding of mHealth’s use during the COVID-19 pandemic in the Philippines.

### Study Design and Sample

A descriptive qualitative design using a combination of individual interviews and focus group discussions (FGDs) was used. Due to the resources available, a total of 16 users of COVID-19–related mHealth technologies were recruited using convenience sampling. Recruitment was conducted through online marketing and referral of stakeholders. Participants were Filipinos, at least 18 years old and not more than 60 years old, residents of the Philippines, and users of any mHealth tool related to the pandemic for at least 1 month. Those who were unable to speak Tagalog or English, as well as those without an internet connection or tools for videoconferencing, were excluded. We initially planned and scheduled 2 FGDs. However, because of the low participant turnout due to scheduling conflicts, we decided to accommodate individual interviews and group discussions following their availability, which resulted in 8 sessions (5 individual interviews and 3 FGDs with 11 participants with whom we have no established relationship or affinity).

### Data Collection

The FGD guide was organized following the 7 topic areas of interest to the research stakeholders: (1) utility, (2) technology readiness level, (3) design, (4) information, (5) usability, (6) features, and (7) security and privacy. These topics reflect the different design and quality criteria for mHealth solutions. The questions were developed based on the Mobile App Rating Scale (MARS) [24]. MARS provides a framework for classifying and assessing the quality of mHealth apps. Examples of questions are shown in Table 1. Data collection happened in February 2022.

The 2 lead authors (AG and RC) served as interviewers, both experienced in qualitative research and with grounded knowledge of digital health. Because of the pandemic, data collection activities were conducted online using a secured and licensed audio- and videoconferencing platform. Each interview or discussion session lasted an average of about 45 minutes to 1 hour, with 1 researcher acting as the interviewer and the other as the notetaker. Furthermore, the interviews or FGDs ended when data saturation was reached.

**Table 1:** Topic areas and examples of questions included in the semistructured questionnaire.

Topic area	Sample question
Utility	Why are you using this mHealth^a^ technology during the pandemic?
Technology readiness level	What do you think are the minimum operating skills of an end user to meaningfully use a COVID-19 mobile technology solution?
Design	How does the technology solution look in general? What are your thoughts on the arrangement and size of elements on the screen?
Information	Do you believe the information that you see in the technology solution and why?
Usability	How easy is it to learn how to use the app?
Features	Can you describe the features that you liked most in the mobile app? What can be improved?
Security and privacy	Do you know and understand how the mobile app deals with your personal and health information?

^a^mHealth: mobile health.

### Data Analysis

Discussion and interview recordings were transcribed and translated into English. Transcripts were not submitted to participants for verification. Rather, they were reviewed by the authors prior to coding using NVivo 12 (QSR International). We adopted a cyclical coding approach [25]. Using this approach, we used the 7 topic areas as domains and initially developed a codebook based on notes during the sessions. Frequent reviews were conducted to discuss and add codes to capture relevant differences and cycled back to ensure that the codes were applied consistently to previously coded transcripts. Following some of the established techniques, including repetition and emphasis, we continued iterating on early potential themes.

### Ethical Considerations

The methods were performed with the approval of and in accordance with the relevant guidelines and regulations of the University of the Philippines Manila Ethics Review Board (UPMREB 2021-472-01) as well as the committee represented by the Philippine Council for Health Research and Development (PCHRD) and the DOH-HTAC.

Informed consent was secured prior to the interviews or FGDs. As agreed, only videoconferencing accounts procured for the study and accounts provided by the University of the Philippines Manila were used for the interviews or FGDs and for initially storing the recordings. Once a recording was available, it was downloaded and saved on a password-protected machine managed by the researchers. The recordings were permanently deleted from the online video platform. No names were included in the transcripts. After the interviews or FGDs, all participants received US $5 as communication allowance for internet connectivity.

## Results

### Characteristics of the Participants

The sociodemographic characteristics of the 16 study participants are summarized in Table 2. There were 6 (37.5%) males and 10 (62.5%) females, and 8 (50.0%) participants were aged 25-35 years, while 2 (12.5%) were above 50 years of age. Most participants had a bachelor's degree (n=8, 50.0%) and worked in the health and wellness sector (n=9, 56.3%). All participants resided in an urban area. In terms of the duration of using mHealth solutions related to COVID-19, a large proportion (n=13, 81.3%) had used mHealth for more than 6 months.

**Table 2:** Demographic characteristics of study participants and their use of mHealth^a^ (N=16).

Sociodemographic variables		Participants, n (%)^b^
**Sex**		
	Male	6 (37.5)
	Female	10 (62.5)
**Age (years)**		
	25-35	8 (50.0)
	36-45	3 (18.8)
	46-50	3 (18.8)
	>50	2 (12.5)
**Professional affiliation**		
	Health and wellness	9 (56.3)
	Manufacturing industry	1 (6.3)
	IT and cyber services	3 (18.8)
	Educational institutions	3 (18.8)
**Education**		
	High school diploma	1 (6.3)
	Some college but no degree	1 (6.3)
	Associate degree	4 (25.0)
	Bachelor's degree	8 (50.0)
	Professional degree	2 (12.5)
**Community/residence**		
	Urban	16 (100)
**Type of mHealth app used**		
	Contact tracing and location tracking	16 (100)
	Health declaration	14 (87.5)
	Symptom tracker	12 (75.0)
	Telehealth/virtual consultation	9 (56.3)
**Duration (months) of using COVID-19 mobile technology solution/app**		
	<1	2 (12.5)
	2-3	1 (6.3)
	>6	13 (81.3)

^a^mHealth: mobile health.

### Description of Themes

The participants noted a range of considerations for the design and quality of mHealth implementation during the COVID-19 pandemic. In total, 18 themes were identified under the 7 focus areas serving as domains and are summarized in Table 3.

**Table 3:** Summary of domains and themes.

Domains	Themes
1. Utility	1.1: Use of mHealth^a^ technologies 1.2: Motivations in using mHealth
2. Technology readiness	2.1: Mobile technology literacy 2.2: User segmentation
3. Design	3.1: User interface design 3.2: Language and content accessibility 3.3: Technology design
4. Information	4.1: Accuracy of information 4.2. Use of information
5. Usability	5.1: Design factors 5.2: Dependency on human processes 5.3: Technical issues
6. Features	6.1: Interoperability and data integration 6.2: Other feature and design recommendations 6.3: Technology features and upgrades
7. Privacy and security	7.1: Trust that mHealth can secure data 7.2: Lack of information 7.3: Policies

^a^mHealth: mobile health.

#### Domain 1: Utility

##### Theme 1.1: Use of mHealth Technologies

The most common mHealth tools used are those designed for contact tracing, location monitoring, and symptom tracking. This is mainly because of policies set by the government that require the collection of data for local and intercity movements, as well as when entering establishments. Several participants pointed out that although they use contact-tracing apps, they only see them as data collection tools. Participants from the health care industry highlighted the use of mHealth for virtual consultation or telehealth.

Despite the COVID-19 situation in the country, I have been in and out of my province since I am a front-line health worker. I use this application for traveling.

Physician, male, 31 years old

##### Theme 1.2: Motivations in Using mHealth

The participants mentioned several motivations for using mHealth technologies during the pandemic. First, they use mHealth because it is mandated by the government or their organization. Second, they want to move around and continue living their lives, for example, traveling for work, entering establishments (eg, markets, shopping malls), and doing virtual medical consultations. One participant even mentioned that for them to receive assistance from their local government, they need to download and use the app. Third, they feel that using mHealth keeps them and others safe. Fourth, it is much more convenient to use mHealth than fill out paper-based forms. Lastly, they trust that using mHealth tools and providing accurate data will help their community improve the restriction status.

Even though I am not that confident with the app, I need that to enter establishments. I feel like I am forced to use it.

Sales agent and collector, female, 41 years old

#### Domain 2: Technology Readiness

##### Theme 2.1: Mobile Technology Literacy

Since the majority of mHealth tools are mobile apps, end users need to be familiar with using a smartphone to navigate across the features of a COVID-19 mobile app. This includes operating skills, such as accessing and downloading from app stores, connecting to Wi-Fi, enabling mobile data, using the camera to scan QR codes, and filling out forms, among others. Although these operating skills may be easy, the participants shared that it took them 1 or 2 days to be comfortable with using the mobile apps.

You need to know how to use a smartphone, connect to the internet, and be familiar with downloading an app.

Call center agent, male, 30 years old

##### Theme 2.2: User Segmentation

Although the participants shared that it did not take that much effort to learn mHealth tools, they recognized that it will be different for every user. They raised the concern that end users who are not technology savvy, including the elderly and those who do not have access to smartphones and the internet, might be disadvantaged. They may take some time to learn and become comfortable using mHealth. Older persons do not have email addresses, a requirement for registration. Moreover, individuals with low literacy may have a difficult time using these technologies.

The older generation is having a hard time using it—our parents who are aged. Older people will have a hard time learning new technology. A jeepney driver who may not have advanced educational attainment [to easily learn it].

Nurse, female, 31 years old

#### Domain 3: Design

##### Theme 3.1: User Interface Design

The participants found the user interface of the mHealth apps to be user friendly and visually appealing. The contents are clear when they have a stable internet connection. Many also described the tools as simple and straightforward—the kind of mHealth tool preferred.

However, a few participants shared that some of the screens are confusing and less intuitive compared to other established mobile apps for banking and in social media. Some prefer simpler screens, bolder fonts, and larger field boxes and buttons.

I don't need a pretty animation. I just need to have a white background and pretty much readable font, that's it.

Special education teacher, male, 42 years old

##### Theme 3.2: Language and Content Accessibility

Although the participants felt comfortable with the design and content language of the mHealth tools, they recommended making them more accessible. First, the contents are in English and there is no option to switch languages; hence, it may be difficult for non-English speakers and those who speak different dialects. Second, participants from the education sector shared that the mobile apps lack accessibility features for people with disabilities (eg, those with visual impairment, low vision, and physical challenges).

The applications require vision to navigate. Those who are visually impaired and those physically challenged would not be able to use it.

Special education teacher, male, 42 years old

##### Theme 3.3: Technology Design

Many of the participants shared their dissatisfaction with the technology design of the mHealth tools. Specifically, mHealth tools are designed for smartphones and require an internet connection. Many are forced to top up mobile data just to use the apps. Not having a stable internet connection makes it difficult to complete the required forms before entering establishments. Those who do not have access to smartphones and internet connections could be denied entry. Fortunately, some establishments provide free Wi-Fi, and some companies offer to pay for mobile data for their workers, but as the participants highlighted, this is not the case for all.

If you don't have mobile internet data or your signal is weak, you can't enter the portal of [the contact tracing app]. You will also not be able to scan the QR code.

Nursing attendant, female, 51 years old

#### Domain 4: Information

##### Theme 4.1: Accuracy of Information

Only a few of the participants trusted the information in the mHealth technologies, particularly the contact-tracing and location-monitoring tools used regularly. Many of them still raised doubts and thought that the information collected by the mHealth tools implemented or endorsed by the government is inaccurate. Because these technologies are simply regarded as data collection tools without verification or use of data, the participants doubted that the information submitted is accurate.

A more positive perspective came from those using mobile apps developed and implemented by their company, mostly private organizations, where the information submitted is verified by security personnel and company clinic staff and the data are secure; hence, they provide accurate information.

I agree that the users can submit inaccurate data. I can lend my phone to someone and let my nieces use the application so they can enter the mall. We've done it before. We realized that it was wrong.

Production supervisor, male, 46 years old

##### Theme 4.2. Use of Information

Even with the continued effort in completing the forms within the contact-tracing and location-monitoring apps, the participants believed that the data are not used. None of them was ever contacted, even though they knew someone who tested positive and was near them. This made them question the real purpose of the mHealth tools. Some participants concluded that they are merely a data collection tool and a requirement to continue moving around during the pandemic.

Maybe [the data from mHealth applications for contact tracing and travel] it's just there. I mean I've been using it for quite some time, and I haven't been contacted, not even once. I doubt no one from all the places I've been [to work as a front-line health care worker] has tested positive for COVID-19.

Physician, male, 31 years old

#### Domain 5: Usability

##### Theme 5.1: Design Factors

The participants agreed that after the learning period, the mobile apps are easier to use. However, there are design factors that alter the end-user experience. For example, the contact-tracing apps promoted by the national government need to be completed and verified every time the users enter an establishment. Other apps simply require presenting QR codes and require less work. Because many of the apps are dependent on an internet connection, the end-user experience and usability are likewise linked on the device as well as the availability and speed of the internet.

It is hard because I must fill out the form again. I also need to put in my cellphone number to confirm that I am indeed the user. The process may take 5-10 minutes before I can enter the establishment or get through the security line.

Call center agent, female, 27 years old

##### Theme 5.2: Dependency on Human Processes

Although the mHealth technologies themselves are usable, the participants acknowledged that they are simply a tool to support a public health measure. In many cases, these mHealth tools are used to collect contact-tracing data and facilitate requests as well as approval of travel. For these 2 use cases, some processes take place outside the mHealth tools. For example, a contact tracer should call an end user if they are exposed. According to the participants, the success and failure of these human-led processes contribute to their perspective on usability.

When I traveled last October back home, I applied for a travel pass [using the mobile application] on a Saturday, and then it got approved on Monday morning and my flight was Monday morning too. I also tried traveling to another province just late last year. I just applied for the pass and never got a response. The status remained pending and never even approved.

Physician, male, 31 years old

##### Theme 5.3: Technical Issues

The participants shared experiences on technical issues affecting the usability of mHealth technologies. Many shared that at one point, their app crashed, encountered problems with registration, gave an error message, and did not function as designed (eg, did not scan the QR code). Many of the participants also said that sometimes the apps are slow. They were unsure whether this was because of the device, app, or the internet connection.

Even though I consider myself as a techie person, when I tried to actually register, I failed many times. There are many technical problems. When your internet is slow and the application will crash in the middle of filling out the form, you have to do it again.

Teacher, female, 44 years old

#### Domain 6: Features

##### Theme 6.1: Interoperability and Data Integration

Many different mHealth tools are designed for the same use case; consolidation was most requested by the participants. For example, since almost every city implemented its own contact-tracing app, they wanted a unified tool or centralized data so they did not have to switch apps in every location. The participants also wanted the apps to integrate data provided to various mHealth tools and systems. Consolidating COVID-19 information, such as travel, vaccine records, and test records, in 1 tool will make it easier for them to provide information, when needed.

I would prefer a national [application] so we don't have different for each local government unit. For example, if I go to a different city, I need to use their mobile app, which is different from what we use in my city. It might be better if we only have 1. It also causes comparison between these technologies.

Special education teacher, male, 42 years old

##### Theme 6.2: Other Feature and Design Recommendations

The participants were satisfied with the current features of the mHealth technologies with additional improvements in the design of the technologies: making the interface more user friendly and simplifying the process. For security, 1 participant recommended the integration of fingerprint validation, which is present in many, but not all, smartphones. Another recommendation was to include updated contact information and where they can get services if they have symptoms or test positive. Many participants also wanted to include more resources to help end users navigate the tool, especially those using it for the first time.

It will be better if they add guides on how to use the application, because not everyone will get [know] how to use it right away.

Call center agent, female, 27 years old

##### Theme 6.3: Technology Features and Upgrades

In terms of the technology, the participants wanted to use the mHealth tools without connecting to the internet, or the LGU or establishment should provide free a Wi-fi connection for patrons. Recommendations to use other types of mobile technology to collect location information were raised. For example, a mobile app can use the location feature of the smartphone to record movement rather than scanning codes through ports or completing forms every time one enters an area. A couple of participants working in the IT field suggested continuously updating the applications as well the servers to address the lag and other technical issues.

I hope that when I enter establishments, I can access the application without the internet. Alternatively, when entering establishments, [a] Wi-fi connection is available for free.

Nursing attendant, female, 49 years old

#### Domain 7: Privacy and Security

##### Theme 7.1: Trust That mHealth Can Secure Data

The participants recognized that the mHealth systems could be hacked. The fear of data exposure is one of the reasons why end users submit inaccurate information. Many of the participants were not confident that the implementers can securely store their data and prevent them from exposure and misuse.

Although there is a general negative perspective on privacy and security, a couple of participants believed that their data are being put into good hands. Although they recognize the risks, they trust the mHealth technologies, and the implementers—that they will keep their data safe.

There will always be the doubt [that my data will be exposed]. However, I choose to trust that the information I share will not be used in a bad way.

Nursing attendant, female, 51 years old

##### Theme 7.2: Lack of Information

Some of the participants remembered having seen the privacy statement in the app, while others did not recall reading it or the consent notice. Many ignored and did not take the time to read the statement. They did not know where their information goes, where it is stored, and who has access to it. They were aware that many apps are developed by a third party contracted by government units or other implementing organizations, making them more anxious about their data.

It is not clear on where my information will be stored, though actually I never read what is in there [privacy/data use statement/agreement].

Call center agent, female, 27 years old

##### Theme 7.3: Policies

The need for balancing policies between requiring the public to share information for public health measures during a pandemic and citizens' rights according to existing laws was highlighted. Some participants believed that during a public health emergency, data privacy should not be the topmost consideration. Although many agreed, they also believed that their data should be protected from unauthorized access, distribution, and use. Some participants from the health sector mentioned that the Data Privacy Act itself is limited and too broad. Therefore, additional standards and protocols on data handling and data destruction should be established.

Policies are needed. If we provide all information to that contact-tracing application, they should be able to secure all those information. Of course, everything is there—address, phone number, full information, even middle name, and birthday.

Nurse, female, 28 years old

## Discussion

### Principal Findings

This study complements the growing body of knowledge that highlights the importance of end-user engagement in designing, implementing, and scaling digital health solutions, including mHealth. Based on the results, we learned that accessibility, privacy and security, simple interface, and integration are some of the design and quality considerations valued by end users.

The results also validate and complement the existing literature. First, although we did not expect accessibility features for those with disabilities to be raised, this is a topic being explored more recently in the field of mHealth [26-29]. The majority are also looking at accessibility for older people, which was also raised by the end users in this study. Second, trust—a repeated concept across themes—has been emphasized as important during a pandemic [30]. Based on the results and contexts, we note negative bias toward mHealth endorsed or implemented by the government. The political landscape in the country and the participants’ localities as well as experience or knowledge of recent data leak incidents could have influenced their perceptions. Lastly, although most of the literature indicates that mHealth can reduce disparities, the thoughts and experiences of the end users reveal otherwise. This qualitative study shows that mHealth design and policies (eg, requiring smartphones and the internet, which is not always accessible) can further exacerbate disparities and exclude certain populations [31-33].

### mHealth Design and Quality Principles

As already discussed, engaging target end users at all stages of the design process is important and results in more useful tools. If mHealth tools do not meet the needs and requirements of the end user, mHealth will likely be misused or underused and eventually fail to meet its original objectives [13].

To advocate and inform user-centric scale-up and future development of mHealth technologies (with a special focus on resource-limited settings), we reflected on the end users’ perspectives and translated them into design and quality principles for mHealth. The principles are summarized in Table 4, together with reference to the themes that point to the challenges or recommendations from end users.

**Table 4:** mHealth^a^ design and quality principles with reference to themes.

Design and quality principles	Theme reference	Example of app
1. mHealth development and implementation should be driven by value.	1.1, 1.2, 4.2, 5.1	A mobile app could start as a contact-tracing app; as the country moves to the postpeak phases, the app can add features to store vaccine records.
2. Interventions using mHealth as a tool should be designed and implemented around inclusion.	2.1, 2.2, 3.3	mHealth should be designed such that it can be used even by old phones, can work without the internet, and should only require minimal digital skills.
3. mHealth should be adapted to cope with users' physical and cognitive restrictions.	3.1, 3.2	Developers can leverage existing accessibility features in mobile operating systems.
4. mHealth design should ensure privacy and security compliance and end-user awareness.	4.1, 7.1, 7.2, 7.3	Consent should be clear and in a language that can easily be understood by its intended users.
5. mHealth should be designed around standards.	6.1	Internally recognized standards should be adopted for mHealth and other digital health systems.
6. mHealth should be trusted by the users.	4.1, 7.1	mHealth should contain accurate content with a reliable source and have secured infrastructure.

^a^mHealth: mobile health.

#### Principle 1: mHealth Development and Implementation Should Be Driven by Value

End users should recognize the value of using mHealth technologies. When they do, it can lead to better utility, voluntary and sustained adoption, and better data. This could be achieved by understanding the needs of the end users and integrating mHealth technology into their respective practices or activities. Although prioritizing what matters to the end users is ideal, the public health measures during a pandemic have certain requirements that need to be followed; mHealth design should balance the 2 concepts [34]. During the different phases of the pandemic, features could be added to keep the end users engaged and recognize its value.

#### Principle 2: Interventions Using mHealth as a Tool Should Be Designed and Implemented Around Inclusion

mHealth is recognized as an important resource for addressing inequalities in health care access [35]. However, this is not always the case. As highlighted in the results, when designed poorly, the use of mHealth can exclude certain populations and further widen inequalities. mHealth, and the public health intervention being supported, should be designed such that all, including those who lack digital skills and motivation, along with having limited or no access to supported devices and connectivity, can get access and benefit from the intervention [36,37]. Developers should consider the situational challenges of end users and provide alternate options to those experiencing challenges (eg, uneducated, inaccessible technologies), such as providing internet access, a mobile kiosk, and paper-based versions.

#### Principle 3: mHealth Should Be Adapted to Cope With Users' Physical and Cognitive Restrictions

Inaccessible user interfaces of digital products and content often led to forms of societal discrimination [38]. Ensuring accessibility in mHealth is important so that individuals with physical and cognitive restrictions can equally benefit from mHealth and gain value from the interventions. In the Philippines, government guidelines for accessibility exist for content on websites [39,40]. Developers can leverage existing frameworks from other countries and well-established mobile accessibility guidelines [41-45]. Developers can also use existing accessibility features in mobile operating systems. For example, both Apple iOS and Google Android provide application programming interface–based services for apps to enable accessibility features, such as guided access and text-to-speech [46,47].

#### Principle 4: mHealth Design Should Ensure Privacy and Security Compliance and End-User Awareness

Despite current efforts, data collection using mHealth technologies by different institutions and establishments raises significant privacy and security challenges [48]. Development and design teams should be compliant with regulatory frameworks, in addition to standard privacy and security practices. End users should feel confident that the tool is secured and that the information is protected and used in accordance with terms and laws. Consent should be clear and in a language understood by many. Developers should also be transparent in data collection, storage, utility, and sharing.

#### Principle 5: mHealth Should Be Designed Around Standards

The lack of guidance during the early phase of the pandemic resulted in siloed development and implementation and, in turn, become a source of confusion, dissatisfaction, and frustration among end users. Whenever available, developers should follow existing guidelines set by government authorities. There are also internationally recognized standards for mHealth and other digital health systems. For example, WHO released technical specifications for the digital documentation of COVID-19 vaccine certificates [49]. Following organizational, semantic, and syntactic standards can help facilitate sharing of vital data between systems and with authorities, if needed, despite users using different mHealth platforms.

#### Principle 6: mHealth Should Be Trusted by the Users

Digital health, including mHealth, relies heavily on trust in order to succeed [50]. Looking at the local mHealth implementation, trust is still generally low. We argue that trust should be addressed both from the technical and from the societal context. First, developers should ensure that mHealth is designed using recognized and evidence-based guidelines, has accurate contents from reliable sources, has secured infrastructure, and delivers its promised outcomes [30]. Second, nontechnical factors, such as disbelief that mHealth technology works, distrust of implementors or keepers of data, and the general fear of data leakage because of recent national events, should be addressed with a societal approach [51] to regain trust.

### Limitations of the Study

This study recognized a number of limitations. Considering its aim, results should not be taken and interpreted as the state of all COVID-19–related mHealth technologies in the Philippines. It was conducted to gather end users’ perspectives to inform future mHealth development. Results should be complemented with other data to fully understand mHealth implementation during the pandemic.

Due to the rapid nature and meager resources, we were able to engage 16 users. Nonetheless, these users provided valuable insights to answer the objectives of the study. Although we believe that we reached saturation, the research could benefit more if the various demographic groups were represented. We recognize that the nonrepresentation of other population groups might have influenced the results. For example, more than half of participants included in this study work in the area of health and wellness. Their views might not represent the majority of users in the Philippines.

The end users also use different mHealth tools. Their inputs may or may not reflect their perspectives on all mHealth technologies. Whenever possible, we included the specific type of mHealth when narrating the feedback from the end users.

### Conclusion

Considerations in the development and implementation of mHealth during a pandemic highlight the importance of engaging end users to ensure that mHealth technologies are designed to address their needs, making it a valuable tool in curbing the worldwide health problem. There is a need to revisit mHealth design and implementation in the Philippines. Developers, researchers, and implementers should consider the 6 principles when scaling up or developing new mHealth solutions. mHealth technologies should be accessible, value driven, secured, trusted, and standardized.

Future studies should focus on assessing mHealth use cases (eg, contact tracing, telehealth, location monitoring) to inform type-specific recommendations. Full-scale evaluation studies should also be conducted to further understand the effectiveness, usability, and quality of mHealth tools.

## Abbreviations

DOH-HTAC: Philippines Department of Health – Health Technology Assessment Council
FGD: focus group discussion
LGU: local government unit
MARS: Mobile App Rating Scale
mHealth: mobile health
WHO: World Health Organization
MARS: Mobile App Rating Scale
